# Seroprevalence of *Toxoplasma gondii* in free-ranging wild boars hunted for human consumption in Estonia

**DOI:** 10.1186/s13028-015-0133-z

**Published:** 2015-08-04

**Authors:** Pikka Jokelainen, Kaisa Velström, Brian Lassen

**Affiliations:** Institute of Veterinary Medicine and Animal Sciences, Estonian University of Life Sciences, Kreutzwaldi 62, 51014 Tartu, Estonia; Faculty of Veterinary Medicine, University of Helsinki, P.O. Box 66, 00014 Helsinki, Finland

**Keywords:** Antibodies, Game, *Sus scrofa*, Toxoplasmosis, Wildlife, Zoonosis

## Abstract

**Background:**

Although the prevalence of human *Toxoplasma gondii* infections is high in Estonia, no information is available on the prevalence of infections in the local animal populations. Wild boars are a good indicator species for estimating the prevalence and spread of *T. gondii* and were thus investigated in this nationwide cross-sectional study. Volunteer hunters sampled cardiac or skeletal muscle of 471 wild boars legally hunted for human consumption in Estonia during the hunting season of 2012–2013. Serosanguineous meat juice samples were obtained from thawed tissue samples, diluted 1:40, and screened for specific anti-*T. gondii* IgG antibodies with a commercial direct agglutination test.

**Results:**

Almost one-quarter (113; 24%) of the wild boars examined were seropositive for *T. gondii*. The seroprevalence did not differ significantly between age groups or sexes. The seroprevalence was lowest in Viljandimaa, which is located in the southern part of Estonia. In other counties, the infection was evenly prevalent.

**Conclusions:**

In Estonia, wild boars are commonly exposed to *T. gondii*, which is endemic and widespread. The consumption of raw or undercooked meat of Estonian wild boars may pose an infection risk to humans and other hosts.

## Background

The meat and other tissues of animals infected with the protozoan parasite *Toxoplasma gondii* can be sources of human infections if eaten undercooked [1, 2]. In many countries, the seroprevalence of *T. gondii* has decreased in the human population [3–6]. By contrast, the seroprevalence remains high in the human population in Estonia, where toxoplasmosis appears to be underestimated. In the human population of Tartu, the seroprevalence of *T. gondii* was 61.8% in 1991–1993 [7] and 54.9% in 1999–2001 [8], and a recent nationwide survey found a seroprevalence of 56.4% [9]. Seropositivity indicates a chronic infection with the parasite, which is not harmless [10–12].

No data are available on the prevalence and spread of *T. gondii* infections in animals hunted or raised for human consumption in Estonia, and the data from neighbouring countries are limited [13]. In this study, we investigated wild boar (*Sus scrofa*), which is a good indicator species for the presence and spread of *T. gondii*. Annually, approximately 20,000 European wild boars are hunted for human consumption in Estonia [14]. This study aimed to estimate the proportion of the hunted wild boars that had a measurable antibody response to *T. gondii*.

## Methods

### Ethics statement

Heart or skeletal muscle of wild boars legally hunted for human consumption and shot by hunters during a hunting season was collected. No animals were killed for the purpose of this study. Wild boar is wild game and not an endangered or protected species in Estonia. The hunters held necessary documents certifying hunting rights, including a hunting certificate, a hunting permit, and a shooting test certificate, issued by the local administrative officials. The hunting followed the local regulations and took place in legal hunting grounds.

The aim of the study and the voluntary nature of the sampling were explained to the hunters. The hunters accepted that submitted samples were used for research purposes. Formal written informed consents were not requested. All information regarding hunters was treated confidentially.

### Study design

The sampling for this nationwide cross-sectional epidemiological study was based on the voluntary contributions of local hunters. Groups of hunters from all 15 counties of Estonia were contacted by a native speaker informing them that if they shoot wild boars, we would be interested to receive samples for this study. More detailed information was sent to the hunters who were interested. The sampling period was one hunting season from 1st October 2012 to 28th February 2013.

### Sample size calculation

The sample size required for an estimate with the desired precision and level of confidence was calculated before sampling using the open source software OpenEpi [15]. The calculation was based on an expected prevalence of the parasite of 30–40%, the rounded limits of the confidence interval of the prevalence reported in free-ranging wild boars in Latvia [13]. The minimum sample size was calculated to be 323–369 samples, and more specifically, by adjusting for the finite number of 20,000 animals to be hunted, the minimum sample size was 318–363 samples.

To collect a proportionally representative sample of the wild boars that are hunted for human consumption in Estonia, the optimum sample size from each county was calculated based on their percentages of the total number of wild boar bagged in the previous year of 2011 [16]. This calculation was performed using an overall sample size of 500 to account for the voluntary nature of sampling and the possible changes in the proportional contributions from the counties to the overall hunting total.

### Collection of samples

The hunters were requested to sample approximately 50 g of the hearts and to place the samples in individual sealable plastic containers. The hunters also provided the background information for each animal, including an estimate of the age, the sex, and the county where the animal was shot. The hunter-harvested samples came from 471 wild boars. The samples were from the hearts of 449 animals, from mixed heart and skeletal muscle of three animals, from muscle other than the heart of 12 animals, and from unspecified muscle of seven animals. For logistical reasons, most of the samples were frozen before or during their shipment to the laboratory, where they were stored frozen at −21°C. Serosanguineous meat juice aliquots were collected when the tissue samples were thawed, and one of the aliquots was used for this study.

### Direct agglutination test

The fluid samples were diluted 1:40 [17, 18] and screened for specific anti-*T. gondii* IgG antibodies with a commercial direct agglutination test (Toxo-Screen DA, bioMérieux, Marcy-l’Étoile, France), following the instructions of the manufacturer. The samples that tested positive at the dilution used were defined as seropositive. The controls that were provided with the kit were included on all plates. The results were read with a light source to avoid any problems with background colour caused by haemolysis.

### Statistical analyses

The estimates of age were categorized into two age groups (up to 1 year or over 1 year). The counties were included in the analyses as dummy variables and as northern and southern counties. Cross tabulations and test statistics (Chi square and Mid-P exact) of the Open Epi software [15] were used to compare results and evaluate associations. Confidence intervals were calculated using Mid-P exact. Differences with *P* values <0.05 were considered statistically significant. The combined effects of the variables on seropositivity were evaluated with logistic regression analyses using the Stata 11.0 software (StataCorp, College Station, TX, USA).

## Results

**Fig. 1:**
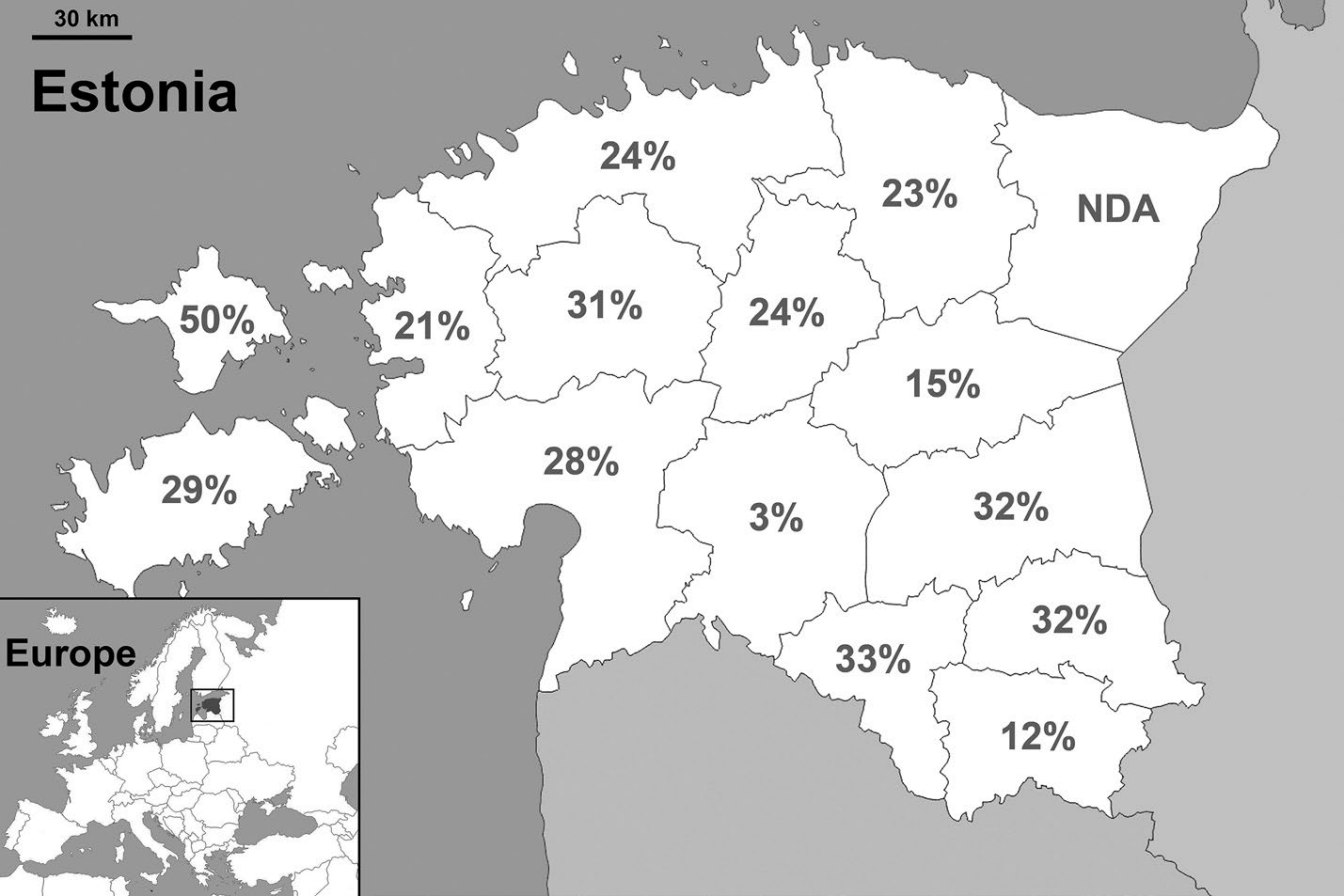
Prevalence of anti-*Toxoplasma gondii* antibodies in wild boars hunted in Estonia, by county (*NDA* no data available).

Of the 471 individual wild boar samples submitted by hunters, 113 (23.99%) were defined as seropositive. The *T. gondii* seroprevalences by county are shown in Fig. 1 and Table 1. The seroprevalence in Viljandimaa was significantly lower (*P* < 0.01) than the overall seroprevalence estimate. In other areas, as the differences were non-significant, the infection seemed to be evenly prevalent. Seroprevalence did not differ significantly between the two age groups or sexes (Table 1). Logistic regression analyses revealed no significant combined effects.

**Table 1:** Prevalence of anti-*Toxoplasma gondii* antibodies in wild boars hunted in Estonia (NDA: no data available)

	% Of the hunting bag	Number of samples^a^	% Of the total sample^a^	Number of positive samples	Seroprevalence (%)	95% Confidence interval
Age group						
Age up to 1 year		156	33.12	35	22.44	16.41–29.48
Older than 1 year		185	39.28	51	27.57	21.49–34.34
Sex						
Female		231	49.04	57	24.68	19.44–30.54
Male		210	44.59	50	23.81	18.42–29.92
County						
Northern counties	40.69	226	47.98	54	23.89	18.67–29.78
Hiiumaa	4.22	4	0.85	2	50.00	9.43–90.57
Läänemaa	7.58	53	11.25	11	20.75	11.43–33.20
Raplamaa	7.62	16	3.40	5	31.25	12.46–56.32
Järvamaa	7.67	37	7.86	9	24.32	12.57–39.97
Harjumaa	6.25	68	14.44	16	23.53	14.60–34.68
Lääne-Virumaa	5.80	48	10.19	11	22.92	12.68–36.66
Ida-Virumaa	1.56	0	0.00	–	NDA	NDA
Southern counties	59.31	245	52.02	59	24.08	19.04–29.73
Võrumaa	5.74	33	7.01	4	12.12	3.97–26.7
Valgamaa	5.04	30	6.37	10	33.33	18.33–51.42
Põlvamaa	7.15	28	5.94	9	32.14	16.95–50.86
Pärnumaa	9.51	46	9.77	13	28.26	16.71–42.47
Saaremaa	15.64	24	5.10	7	29.17	13.74–49.36
Viljandimaa	7.64	30	6.37	1	3.33^†^	0.17–15.36
Tartumaa	4.85	41	8.70	13	31.71	18.91–47.01
Jõgevamaa	3.74	13	2.76	2	15.38	2.66–42.23
Total		471		113	23.99	20.30–28.01

^a^For some samples, the age group of the animal, sex of the animal, or the county where the animal was shot was not reported or not clearly indicated. Thus, these samples were excluded from the respective analyses.

^†^Significantly lower than the overall estimate.

## Discussion

The European wild boar is a popular game species in many countries, and its meat can be a source of human infection with *T. gondii*. Based on the serological evidence gathered in this study, wild boars commonly encountered *T. gondii* in Estonia. As free-ranging animals, wild boars do not acknowledge country borders, and therefore the results can be cautiously considered to represent a wider area of north-eastern Europe.

A recent estimate of *T. gondii* seroprevalence in free-ranging wild boars in the southern neighbouring country, Latvia, was 35.1% [13], which is significantly higher than that found in this study (*P* < 0.001). Despite the two studies are not directly comparable because different methodologies were employed, a geographical gradient in seroprevalence may exist in Baltic countries. In Finland, which is located across the Baltic Sea north of Estonia, a gradient was evident as higher *T. gondii* seroprevalences in both wild and domestic animals, including farmed wild boar, were detected in the southwestern parts of Finland [17, 18].

The high seroprevalence in wild boars and the even distribution of seropositive animals across counties show that *T. gondii* is endemic, common, and likely sustained by homogenous infection pressure in Estonia. One area, Viljandimaa, had significantly lower seroprevalence than the overall level of seroprevalence. The reason for this is unclear. In other areas, the infection appeared evenly distributed and common. However, this study was designed to estimate the overall level of seroprevalence among hunted wild boars and was not designed to reveal differences at a county level.

The number of samples exceeded the precalculated sample size and was thus sufficient to estimate the overall level of seroprevalence. The samples were taken from wild boars that were hunted for human consumption, and the study included animals from 14 of the 15 counties in Estonia; a proportionally representative sample was partially achieved. The contribution of each county to the overall wild boar hunt of 2012 [14] and the distribution of samples taken from wild boars in each county are shown in Table 1. The relatively low number of samples from the islands Hiiumaa and Saaremaa might have been the result of logistical challenges. The failure to obtain any samples from Ida-Virumaa may have been because the area is largely Russian speaking, and the hunters were queried in Estonian language. This lack of samples from the most northeastern part of the country was expected to have only a minor effect on the overall estimate of seroprevalence because the local hunt size was less than 2% of the total.

Background information for each sample was provided by the hunters and was incomplete for some samples. Because the sampling was hunter-harvested and took place during the winter, no samples were obtained from piglets. The determination of the exact ages of the animals was not feasible and some estimates of age made by hunters were excluded from the analyses. The estimates of age were critically evaluated and the animals were categorized into two age groups. Seroprevalence was not significantly different between the two age groups. Assuming the antibodies persisted after infection, older animals would be expected to have higher seroprevalence than younger animals. However, the lifelong persistence of antibodies in wild boars has been questioned [19]. Negative test results for older animals might not exclude that the animals encountered the parasite earlier in their life, and thus the prevalence of the infection in the older age group might have been underestimated. Nevertheless, many wild boars had encountered the parasite during their first year of life, indicating high infection pressure in Estonia.

The estimate of seroprevalence is conservative for methodological reasons as well. Only one class of antibodies was examined, and the cut-off for seropositivity was higher than that used in several other studies [20–22]. The cut-off was selected to yield cautious estimate of the seroprevalence and comparable data with the Finnish study [17]; the prevalence of the infection is probably underestimated. Some false negative results might have been a consequence of the prozone phenomenon, which occurs when antibodies are present in excess for the test. This phenomenon is not detectable when only one dilution of the sample is tested. Only one dilution was used in this study because the little additional benefit expected from using several dilutions would not have outweighed the costs [18].

Sample quality was affected by the sampling method and the logistics of obtaining samples during the winter hunting season. Although we advised that only heart muscle samples should be collected, some of the samples turned out to be from other muscles, despite the fact that heart muscle sampling is relatively easy. Many samples were frozen before or during their transport to the laboratory, and all were put into a freezer upon arrival in the laboratory. Thus, some samples were frozen twice. As a result, haemolysis was observed in fluid samples, but we consider this was unlikely to have affected the results. Although the method used in this study, the direct agglutination test, has only been validated using good quality human sera, it has been widely used in animal studies [20]. The method has been used for fluid samples other than sera, and haemolysis does not affect the results [18, 20, 22].

The infections were naturally acquired, but the sources of the infections remain unknown. Wild boars are considered a good indicator species for environmental contamination with *T. gondii* because they are presumed to acquire the infection from contact with soil. However, wild boars are omnivorous animals and could be infected with all three infective forms of the parasite: sporozoites of oocysts that have sporulated in the environment after having been shed there in unsporulated form by felids; bradyzoites of tissue cysts, which are carried in the tissues of chronically infected hosts; and tachyzoites, which dominate in acute infections and may cause transplacental [23] and possibly galactogenic and venereal infections.

No clinical signs of toxoplasmosis were reported in the wild boars. All the wild boars were apparently healthy, and the chronic infections were subclinical. However, toxoplasmosis can be an animal health and welfare issue. The clinical signs reported during *T. gondii* outbreaks in domestic pigs include high fever, anorexia, dyspnea, vomiting, weakness, recumbency, abortions, and death [24]. Congenital toxoplasmosis with prognathism, oronasal communication, agenesis of nasal cartilage, and bilateral ocular agenesis has been reported in wild boars [23].

The wild boars examined in this study were all hunted and presumably used for human consumption. The current meat inspection does not attempt to detect *T. gondii*, and the meat from game animals is often consumed in the households of hunters without inspection. Serology does not detect the parasite directly, but measures only the immunological response of the host to the parasite. However, for *T. gondii*, seropositivity has been shown to correlate well with chronic infection in pigs [25]. In chronic infection, the parasite remains in the tissues of the host. Furthermore, *T. gondii* parasites have been isolated from seropositive wild boars, including many with lower titers of antibodies than our cut-off for seropositivity [21, 26]. Our result thus suggests that a substantial proportion of wild boars that were hunted in Estonia carried infectious tissue cysts.

## Conclusions

Wild boars commonly encounter *T. gondii* in different parts of Estonia, many during their first year of life. This indicates that the parasite is common, endemic and widespread in the country. Because seropositivity correlates with chronic infection, the consumption of raw or undercooked wild boar meat from Estonia poses a health risk for humans and other hosts of *T. gondii*.
